# Prevention of alcohol withdrawal seizure recurrence and treatment of other alcohol withdrawal symptoms in the emergency department: a rapid review

**DOI:** 10.1186/s12873-021-00524-1

**Published:** 2021-11-06

**Authors:** Justin Jek-Kahn Koh, Madeline Malczewska, Mary M. Doyle-Waters, Jessica Moe

**Affiliations:** 1 Addiction Medicine Fellowship Program, British Columbia Centre for Substance Use, Vancouver, BC Canada; 2 Royal College Emergency Medicine Residency Program, Department of Emergency Medicine, College of Medicine, University of Saskatchewan, Saskatoon, SK Canada; 3 Faculty of Medicine, University of British Columbia, Vancouver, BC Canada; 4 Centre for Clinical Epidemiology and Evaluation, Vancouver Coastal Health Research Institute, Vancouver, BC Canada; 5 Department of Emergency Medicine, University of British Columbia, Vancouver, BC Canada; 6 Department of Emergency Medicine, Vancouver General Hospital, Vancouver, BC Canada; 7 British Columbia Centre for Disease Control, Vancouver, BC Canada

**Keywords:** Alcohol, Substance use, Emergency department, Drug therapy

## Abstract

**Background:**

Patients who experience harms from alcohol and other substance use often seek care in the emergency department (ED). ED visits related to alcohol withdrawal have increased across the world during the COVID-19 pandemic. ED clinicians are responsible for risk-stratifying patients under time and resource constraints and must reliably identify those who are safe for outpatient management versus those who require more intensive levels of care. Published guidelines for alcohol withdrawal are largely limited to the primary care and outpatient settings, and do not provide specific guidance for ED use. The purpose of this review was to synthesize published evidence on the treatment of alcohol withdrawal syndrome in the ED.

**Methods:**

We conducted a rapid review by searching MEDLINE, Embase, and the Cochrane Central Register of Controlled Trials (1980 to 2020). We searched for grey literature on Google and hand-searched the conference abstracts of relevant addiction medicine and emergency medicine professional associations (2015 to 2020). We included interventional and observational studies that reported outcomes of clinical interventions aimed at treating alcohol withdrawal syndrome in adults in the ED.

**Results:**

We identified 13 studies that met inclusion criteria for our review (7 randomized controlled trials and 6 observational studies). Most studies were at high/serious risk of bias. We divided studies based on intervention and summarized evidence narratively. Benzodiazepines decrease alcohol withdrawal seizure recurrence and treat other alcohol withdrawal symptoms, but no clear evidence supports the use of one benzodiazepine over another. It is unclear if symptom-triggered benzodiazepine protocols are effective for use in the ED. More evidence is needed to determine if phenobarbital, with or without benzodiazepines, can be used safely and effectively to treat alcohol withdrawal in the ED. Phenytoin does not have evidence of effectiveness at preventing withdrawal seizures in the ED.

**Conclusions:**

Few studies have evaluated the safety and efficacy of pharmacotherapies for alcohol withdrawal specifically in the ED setting. Benzodiazepines are the most evidence-based treatment for alcohol withdrawal in the ED. Pharmacotherapies that have demonstrated benefit for treatment of alcohol withdrawal in other inpatient and outpatient settings should be evaluated in the ED setting before routine use.

## Background

### Rationale

Patients who experience harms from alcohol and other substance use often seek care in the emergency department (ED) [1, 2]. In recent years, ED visits related to alcohol in North America have increased significantly [3, 4]. In the United States, the rate of acute alcohol-related ED visits increased 40% between 2006 and 2014. Although national-level data are lacking in Canada, a provincial-level analysis in Ontario demonstrated that between 2003 to 2016, the increase in rates of alcohol-related visits was 4.4 times greater than the increase in all-cause ED visits [3].

In 2016, alcohol use was the seventh-leading risk factor for deaths and disability-adjusted life years globally [5]. Among heavy alcohol users admitted for hospital management, the incidence of alcohol withdrawal syndrome is estimated to be 1.9 to 6.7% [6]. Many patients with alcohol use disorder will require management of alcohol withdrawal during their ED visit [7, 8]. Alcohol withdrawal in the ED has been associated with increased use of critical care resources [9], and frequent ED visits for alcohol-related presentations have been associated with high relative mortality rates [1, 10]. Furthermore, recent studies have demonstrated significant increases in the proportion of alcohol withdrawal-related ED visits during the COVID-19 pandemic, highlighting the rapidly growing burden of alcohol use on EDs across the world [11–14].

ED clinicians are responsible for risk-stratifying patients with alcohol withdrawal syndrome under time and resource constraints, and must reliably identify those who are safe for outpatient management versus those who require more intensive levels of care [7]. Published clinical guidelines recommend stratifying patients with alcohol withdrawal based on their risk of developing complications (e.g., generalized tonic-clonic seizures and delirium tremens) [15–18]. These guidelines are largely limited to the primary care and outpatient settings and do not provide specific guidance for ED clinicians [15–17].

Although one recent literature review summarized evidence for ED withdrawal management, the authors extrapolated recommendations from guidelines for non-ED settings [19]. Another recent literature review also included evidence from non-ED studies [20]. Given the ongoing rise in ED visits due to alcohol withdrawal, and further exacerbation during the current COVID-19 pandemic, there is an urgent need to determine whether certain clinical interventions, when initiated in the ED, could reduce the need for admission and/or prevent other complications of alcohol withdrawal.

### Objectives

Our main objective was to synthesize evidence from published studies on the treatment of alcohol withdrawal syndrome among adult ED patients.

## Methods

In this rapid review, we adapted traditional systematic review methods to generate evidence within an accelerated time frame [21–23]. Rapid reviews are a pragmatic and resource-efficient approach to knowledge synthesis that remains scientific, transparent and reproducible [24]. The utility and importance of rapid reviews is recognized by the Cochrane Rapid Review Methods Group [25], and health policy institutions such as the World Health Organization and the Canadian Agency for Drugs and Technologies in Health [26, 27].

We prepared this paper in accordance with the 2009 Preferred Reporting Items for Systematic Reviews and Meta-Analyses (PRISMA) checklist [28]. Our adaptations to allow rapid review were that one reviewer performed title/abstract screening and quality assessments rather than two independent reviewers performing these steps in duplicate. Our search strategy utilized focused search terms in the most highly relevant databases to prioritize yielding citations with greatest relevance.

### Protocol and registration

In line with our goal of producing this evidence summary expeditiously, we did not publish a review protocol or register this review prior to study initiation.

### Eligibility criteria

#### Population

Adult patients (18 years and older) who presented to the ED with any clinical feature of alcohol withdrawal syndrome, as determined by criteria specified by study authors, e.g., Clinical Institute Withdrawal Assessment for Alcohol (CIWA) score.

#### Intervention

Any clinical intervention aimed at treating alcohol withdrawal symptoms, signs, or complications; and administered via any route. We excluded studies that examined psychosocial interventions alone, or supportive interventions alone.

#### Outcome

Any clinical or patient-oriented outcome related to alcohol withdrawal.

#### Study design

Interventional studies with or without a comparator group, including randomized controlled trials (RCT) and non-randomized trials, as well as observational cohort studies that evaluated an intervention. We excluded review articles and case reports, studies published prior to 1980, non-English publications, and non-human studies.

### Information sources

We searched MEDLINE, Embase, and Cochrane Central Register of Controlled Trials (CENTRAL) from 1980 to 2020 through Ovid. All three databases were last searched on May 11, 2020.

A professional health sciences librarian (MDW) developed our search strategy. We searched MEDLINE and CENTRAL using concepts *emergency department AND alcohol withdrawal AND (drugs OR drug subheadings)*. We searched EMBASE using concepts *emergency department AND alcohol withdrawal* (focused) AND (drugs OR drug subheadings), as well as *concepts emergency department AND alcohol withdrawal AND drugs AND drug subheadings.* Subheadings and keywords were included in the searches to increase sensitivity.

We performed the grey literature search using the search engine Google using combinations of terms [“emergency department”, “emergency room”, “emergency” or “accident and emergency”] and [“alcohol withdrawal”] and [“treatment” or “intervention” or “management”]. The first 50 search results were opened and reviewed for relevant materials. We also hand-searched the most recent conference abstracts (2015 to 2020) of the Canadian Society of Addiction Medicine, American Society of Addiction Medicine, Canadian Association of Emergency Physicians, and American College of Emergency Physicians.

### Search

We report our full electronic search strategy for MEDLINE (Ovid) in Appendix 1.

### Study selection

A single reviewer (MM) performed title and abstract screening. Before full-text screening, articles were flagged for secondary review by the principal investigator (JM) as needed. Inclusion and exclusion decisions for full-text articles were performed in duplicate by two trained reviewers (MM and JK).

### Data collection process

Data extraction was performed independently and in duplicate by two extractors (MM and JK). The principal investigator (JM) arbitrated and resolved any issues that arose during data extraction.

### Data items

We extracted information relating to the study design and characteristics, and results as follows:

#### Study characteristics

authors, year of publication, study design, study location, study time period (start and end dates), follow-up period (if applicable), data sources.

#### Study participants

inclusion and exclusion criteria, age, sex, ethnicity, alcohol withdrawal severity at presentation, method of determining alcohol withdrawal, comorbidities, number of participants in main analysis, losses to follow-up.

#### Study intervention

method of allocation, method of determining eligibility for intervention, description of intervention (type, duration, dose, and timing), person administering intervention, other components of the intervention, method of determining end-point, components of the intervention after ED visit, follow-up after ED visit.

#### Study outcomes

person ascertaining outcomes, primary outcomes, secondary and tertiary outcomes, adverse events.

### Risk of bias in individual studies

We used the Cochrane risk-of-bias tool for randomized trials Version 2 (RoB 2) to assess the risk of bias in the RCTs included in this study [29]. For non-randomized studies, we used the Cochrane risk of bias in non-randomized studies of interventions (ROBINS-I) tool [30]. Risk of bias assessments were performed by one trained reviewer (JK) and verified by the principal investigator (JM).

### Synthesis of results

Due to clinical and methodological heterogeneity of included RCTs, we did not meta-analyze their results. Instead, we present a narrative summary of the results of all included studies.

## Results

### Study selection

**Fig. 1:**
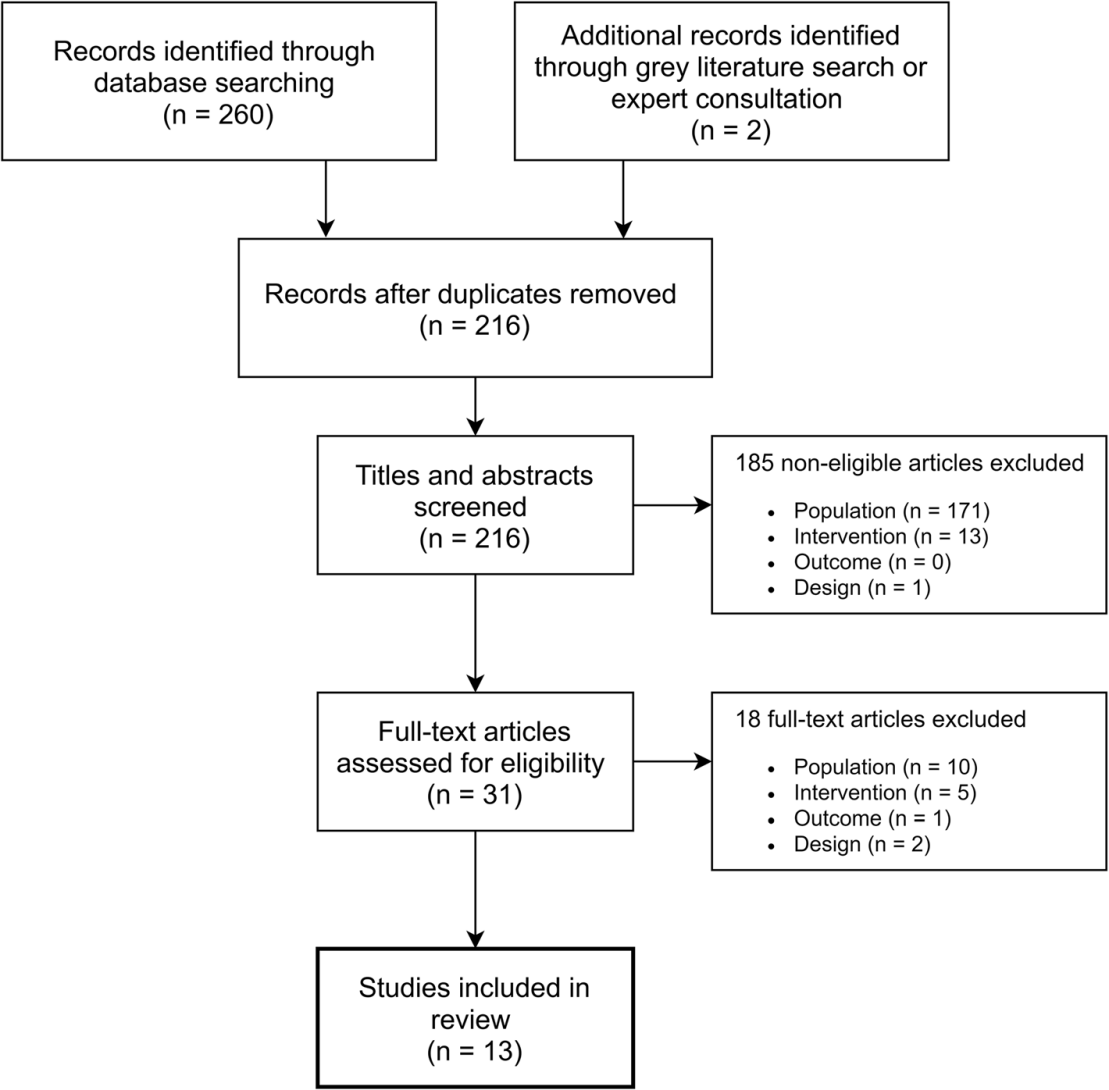
Evidence search and selection

Our search retrieved a total of 214 references after 46 duplicates were removed from searches in health databases. Two other papers were found through grey literature searches. Following title/abstract and full-text inclusion screens, we identified 13 studies that met inclusion criteria for our review. The study flow diagram is displayed in Fig. 1.

### Study characteristics

There were seven RCTs, two retrospective cohort studies, and four retrospective chart reviews. Publication dates ranged from 1989 to 2020. Study characteristics are displayed in Table 1.

**Table 1:** Study characteristics (grouped by intervention)

Author	Year	Location	Study design	*N*
**Benzodiazepines alone**				
*Benzodiazepines* vs. *placebo*				
D’Onofrio G, Rathlev NK, Ulrich AS, Fish SS, Freedland ES [31]	1999	United States	Randomized controlled trial	186
Naranjo CA, Sellers EM, Chater K, Iversen P, Roach C, Sykora K [32]	1983	Canada	Randomized controlled trial	41
*Benzodiazepine selection*				
Scheuermeyer FX, Miles I, Lane DJ, Grunau B, Grafstein E, Sljivic I, et al. [32]	2020	Canada	Retrospective chart review	898
*Symptom-triggered therapy*				
Ismail MF, Doherty K, Bradshaw P, O’Sullivan I, Cassidy EM [33]	2019	Ireland	Retrospective chart review	174
Cassidy EM, O’Sullivan I, Bradshaw P, Islam T, Onovo C [35]	2012	Ireland	Retrospective chart review	99
**Combined phenobarbital and benzodiazepines**				
Ibarra Jr. F [36]	2020	United States	Retrospective chart review	78
Sullivan SM, Dewey BN, Jarrell DJ, Vadiei N, Patanwala AE [34]	2019	United States	Retrospective cohort study	209
Rosenson J, Clements C, Simon B, Vieaux J, Graffman S, Vahidnia F, et al. [35]	2013	United States	Randomized controlled trial	198
**Phenobarbital alone**				
Nelson AC, Kehoe J, Sankoff J, Mintzer D, Taub J, Kaucher KA [36]	2019	United States	Retrospective cohort study	300
Hendey GW, Dery RA, Barnes RL, Snowden B, Mentler P [40]	2011	United States	Randomized controlled trial	44
**Phenytoin alone**				
Rathlev NK, D’Onofrio G, Fish SS, Harrison PM, Bernstein E, Hossack RW, et al. [37]	1994	United States	Randomized controlled trial	100
Chance JF [42]	1991	United States	Randomized controlled trial	55
Alldredge BK, Lowenstein DH, Simon RP [38]	1989	United States	Randomized controlled trial	90

### Risk of bias within studies

Among the seven RCTs, one was at high risk of bias, two had some concerns, and the remainder was at low risk. All non-randomized studies were at serious risk of bias. Table 2 lists the risk of bias assessments for the studies we summarized.

**Table 2:** Main outcomes, key findings, and risk of bias assessments for summarized studies

Author	Participants	Intervention	Comparison	Main outcome(s)	Key results			Risk of bias
***Benzodiazepines alone***					***Intervention group***	***Comparison group***		
*Benzodiazepine* vs. *placebo*								
D’Onofrio G, Rathlev NK, Ulrich AS, Fish SS, Freedland ES (1999) [31]	Adults with witnessed generalized seizure related to alcohol	Lorazepam 2 mg IV (n = 100)	Placebo (*n* = 86)	Seizure recurrence within 6 h of intervention	3/100 (3.0%) *	21/86 (24.4%)odds ratio 10.4 (95% CI: 3.6 to 30.2)		Low
Naranjo CA, Sellers EM, Chater K, Iversen P, Roach C, Sykora K (1983) [39]	Adults with mild-to-moderate AWS (clinician assessment)	Scheduled lorazepam 2 mg PO q2h (max. 3 doses) (*n* = 21)	Placebo (n = 20)	Seizure recurrence within 6 h of intervention	1/21 (4.8%) *	3/20 (15.0%)		Low
*Benzodiazepine selection*								
Scheuermeyer FX, Miles I, Lane DJ, Grunau B, Grafstein E, Sljivic I, et al. (2020) [32]	Adults with AWS (ED discharge diagnosis) treated with lorazepam or diazepam	Lorazepam (dose and route at physician discretion) (*n* = 394)	Diazepam (dose and route at physician discretion) (*n* = 504)	1. Hospital admission (including ICU)	69/394 (17.5%) *effect size*: − 1.2 (95% CI: − 6.3 to 4.2)	94/504 (18.7%)		Serious
				2. Seizures in ED before treatment	3/394 (0.8%) *effect size: −0.03 (95% CI: −1.7 to 1.5)	4/504 (0.8%)		
				3. ED length of stay (non-admitted patients)	Median 266 min (IQR 163 to 387)*effect size:* −33 (95% CI: −75 to −6)	Median 299 min (IQR 192 to 463)		
*Symptom-triggered therapy*								
Ismail MF, Doherty K, Bradshaw P, O’Sullivan I, Cassidy EM (2019) [33]	Adults with AWS (clinician assessment) placed on treatment protocol in a short stay clinical decision unit	Symptom-triggered diazepam (route not specified) when CIWA ≥10 (*n* = 174)	N/A	1. Cumulative diazepam dose	Median 20 mg (IQR 80)	N/A		Serious
				2. Duration of symptom-triggered protocol	Median 12 h (IQR 12)			
				3. Length of stay in clinical decision unit	Median 22 h (IQR 20)			
				4. ED discharge	169/174 (97.1%)			
Cassidy EM, O’Sullivan I, Bradshaw P, Islam T, Onovo C (2012) [40]	Adults with AWS (clinician assessment) treated in the ED clinical decision unit	Symptom-triggered benzodiazepine (*n* = 49)	Fixed dose benzodiazepine (n = 50)	1. Cumulative benzodiazepine dose (in diazepam equivalents)	Median 80 mg (range 0 to 900) *	Median 170 mg (range 15 to 720)		Serious
				2. Hospital length of stay	Median 2 days (range 1 to 9) *	Median 3 days (range 1 to 12)		
***Combined phenobarbital and benzodiazepines***								
Ibarra Jr. F (2020) [41]	Adults with moderate/severe AWS requiring treatment (clinician assessment)	Phenobarbital 130 to 260 mg IV + symptom-triggered lorazepam PO/IV (*n* = 40)	Symptom-triggered lorazepam PO/IV (*n* = 38)	1. Total lorazepam doses (Day 1)	Median 16 mg (IQR 6 to 32)	Median 10 mg (IQR 6 to 19)		Serious
				2. Total lorazepam doses (Day 2)	Median 10 mg (IQR 2 to 29)	Median 6 mg (IQR 2 to 12)		
				3. Total lorazepam doses (Day 3)	Median 2 mg (IQR 0 to 30)	Median 2 mg (IQR 0 to 6)		
				4. ED discharge	4/40 (10.0%)	2/38 (5.3%)		
				5. Hospital admission (non-ICU)	34/40 (85.0%)	32/38 (84.2%)		
				6. ICU admission	2/40 (5.0%)	4/38 (10.5%)		
				7. Discharged within three days of admission	9/40 (22.5%)	2/38 (5.3%)		
Sullivan SM, Dewey BN, Jarrell DJ, Vadiei N, Patanwala AE (2019) [34]	Adults with primary ED diagnosis of AWS	Phenobarbital +/− symptom-triggered benzodiazepine (*n* = 97)	Symptom-triggered benzodiazepine (*n* = 112)	1. ICU admission	14/97 (14.4%)	12/112 (10.7%)		Serious
				2. ED length of stay	Median 9 h (IQR 6 to 14)	Median 9 h (IQR 6 to 14)		
				3. Median hospital length of stay	3 days (IQR 2 to 5)	4 days (IQR 2 to 6)		
				4. Hospital admission (non-ICU)	41/97 (42.3%)	60/112 (53.6%)		
				5. ED discharge	42/97 (43.3%)	40/112 (35.7%)		
				6. CIWA scores at ED discharge	Median 7 (IQR 4 to 12)	Median 7 (IQR 4 to 14)		
Rosenson J, Clements C, Simon B, Vieaux J, Graffman S, Vahidnia F, et al. (2013) [35]	Adults with suspected AWS (clinician assessment)	Phenobarbital 10 mg/kg IV over 30 min + symptom-triggered lorazepam PO/IV (n = 100)	Symptom-triggered lorazepam PO/IV (*n* = 98)	1. ICU admission	4/51 (7.8%)*effect size*: 17 (95% CI 4 to 32)	13/51 (25.5%)		High
				2. Telemetry unit admission	23/51 (45.1%)*effect size*: −6 (95% CI −25 to 13)	20/51 (39.2%)		
				3. General ward admission	24/51 (47.1%)*effect size*: −12 (95% CI −31 to 7)	18/51 (35.3%)		
				4. Hospital length of stay (non-ICU)	Median 76 h (IQR 54 to 114)*effect size*: 42 (95% CI −4 to 82)	Median 118 h (IQR 47 to 190)		
				5. ICU length of stay	Median 34 h (IQR 30 to 276) *effect size*: 60 (95% CI − 170 to 434)	Median 94 h (IQR 43 to 134)		
***Phenobarbital alone***								
Nelson AC, Kehoe J, Sankoff J, Mintzer D, Taub J, Kaucher KA (2019) [36]	Adults requiring medical treatment for AWS (clinician assessment)	Phenobarbital IV (n = 100)	1. Diazepam IV (n = 100)2. Phenobarbital IV + lorazepam IV (*n* = 100)	1. ICU admission	13/100 (13.0%)	Diazepam: 8/100 (8.0%)Phenobarbital + lorazepam: 11/100 (11.0%)		Serious
				2. Hospital admission (non-ICU)	41/100 (41.0%)	Diazepam: 27/100 (27.0%)Phenobarbital + lorazepam: 36/100 (36.0%)		
				3. Hospital length of stay (non-ICU)	96 h	Diazepam: 137 hPhenobarbital + lorazepam: 71 h		
Hendey GW, Dery RA, Barnes RL, Snowden B, Mentler P (2011) [42]	Adults with known or suspected AWS (clinician assessment)	Phenobarbital 260 mg IV (initial dose) + 130 mg IV (subsequent doses) repeated at physician discretion (*n* = 25)	Lorazepam 2 mg IV (initial dose) + 2 mg IV (subsequent doses) repeated at physician discretion (*n* = 19)	1. Change in CIWA score (from baseline to ED discharge)	−9.6	−12.6		Some concerns
				2. ED length of stay	267 min	256 min		
				3. Hospital admission	12/25 (48.0%)	16/19 (84.2%)		
***Phenytoin alone***								
Rathlev NK, D’Onofrio G, Fish SS, Harrison PM, Bernstein E, Hossack RW, et al. (1994) [37]	Adults with alcohol withdrawal seizure	Phenytoin 15 mg/kg IV over 20 min (*n* = 49)	Normal saline placebo (*n* = 51)	Post-infusion seizure recurrence within 6 h	10/49 (20.4%)*effect size*: 3 (95% CI: − 16 to 16)	12/51 (23.5%)		Low
Chance JF (1991) [43]	Adults with alcohol withdrawal seizure	Phenytoin 15 mg/kg IV (maximum dose 1000 mg, maximum rate 37 mg/min) (*n* = 28)	Normal saline placebo (*n* = 27)	Post-infusion seizure recurrence within 6 h	6/28 (21.4%)*effect size*: 2 (95% CI: − 20 to 16)	5/27 (18.5%)		Low
Alldredge BK, Lowenstein DH, Simon RP (1989) [38]	Adults with alcohol withdrawal seizure	Phenytoin 1000 mg IV over 20 min (*n* = 45)	Normal saline placebo (*n* = 45)	Post-infusion seizure recurrence within 12 h	6/45 (13.3%)*effect size*: 0 (95% CI: − 14 to 14)	6/45 (13.3%)		Some concerns

* statistically significant difference between groups (*p* < 0.05)

AWS = a*lcohol withdrawal syndrome*

CIWA = *Clinical Institute Withdrawal Assessment for Alcohol*

ED = *emergency department*

ICU = *intensive care unit*

IV = *intravenous*

### Results of individual studies

Table 2 also lists the main outcomes and key findings for the studies we summarized. We reported effect sizes whenever available.

### Synthesis of results

#### Benzodiazepines alone

##### Benzodiazepines vs. placebo

Two RCTs published before 2000 compared the use of benzodiazepines vs. placebo [31, 39]. One RCT (*n* = 186) of adults with witnessed generalized seizures found that normal saline placebo resulted in a significantly higher risk of recurrent seizure within six hours when compared to a single 2 mg dose of IV lorazepam (odds ratio 10.4, 95% CI: 3.6 to 30.2) [31]. Another RCT (*n* = 41) of patients in mild-to-moderate alcohol withdrawal (without medical complications or witnessed seizures) reported greater proportions of patients with improvement in withdrawal scores (defined as CIWA score ≤ 10) between groups who were allocated to sublingual lorazepam compared to sublingual placebo (95.2% vs. 85.0%, *p* < 0.001) [39].

##### Benzodiazepine selection

One retrospective chart review (*n* = 898) conducted in multiple EDs in Vancouver, Canada compared patients who received lorazepam versus diazepam as their initial management in the ED. [32] Initial CIWA scores were similar between groups (median 17 [IQR 13 to 22]). They reported no differences in terms of hospital admission (− 1.2, 95% CI: − 6.3 to 4.2). Of note, physicians in the study were free to select their choice of benzodiazepine and route of administration.

##### Symptom-triggered therapy

Two studies reported results of a symptom-triggered protocol implemented in an ED clinical decision unit [33, 40]. One descriptive retrospective chart review (*n* = 174) reported that patients placed on a symptom-triggered benzodiazepine protocol received a median cumulative diazepam dose of 20 mg (IQR 80 mg), and 97.1% were ultimately discharged from the ED, although they did not have a comparison group [33]. Another retrospective chart review (*n* = 99) compared those placed on a CIWA-based symptom-triggered protocol with a non-matched comparison group that received a standard tapered benzodiazepine regime [40]. The symptom-triggered group received lower cumulative benzodiazepine doses (median 80 mg vs. 170 mg, *p* = 0.000), and had shorter lengths of stay (median 2 days vs. 3 days, *p* = 0.006).

#### Combined phenobarbital and benzodiazepines

In one retrospective chart review (*n* = 78), patients who received a single IV dose of phenobarbital (130 to 260 mg) in the ED with symptom-triggered lorazepam compared to those who only received symptom-triggered lorazepam had no statistically significant differences in terms of ED discharges (10.0% vs. 5.3%, *p* = 0.43), hospital admissions (85.0% vs. 84.2%, *p* = 0.92), or intensive care unit (ICU) admissions (5.0% vs. 10.5%, *p* = 0.36) [41].

In another retrospective cohort study (*n* = 209), the phenobarbital and benzodiazepine groups had similar proportions of ICU admission (14.4% vs. 10.7%, *p* = 0.53), ED length of stay (9 h vs 9 h, *p* = 0.048), and CIWA scores at ED discharge (7 vs. 7, *p* = 0.32) [34]. Of note, 81% of the phenobarbital group also received benzodiazepines. The two groups did not differ significantly in terms of complications, such as intubation and seizure.

Finally, one RCT (*n* = 198) compared a single dose of phenobarbital (10 mg/kg IV over 30 min) followed by symptom-triggered lorazepam (oral/IV) versus symptom-triggered lorazepam only [35]. The phenobarbital group had significantly fewer ICU admissions than the placebo group (7.8% vs. 25.5%, difference 17% [95% CI: 4–32]), but there was no difference in adverse outcomes (intubation, seizure, use of mechanical restraints, and need for bedside sitter).

#### Phenobarbital alone

One retrospective cohort study (*n* = 300) compared phenobarbital alone with two different protocols: 1) IV diazepam alone, and 2) combined IV phenobarbital and IV lorazepam [36]. The three protocols were performed in different time periods and were the result of medication shortages. There were no differences between the three groups in the primary outcome of ICU admission (13.0, 8.0, and 11.0% respectively, *p* = 0.99).

Only one RCT (*n* = 44) compared phenobarbital alone versus benzodiazepines [42]. Patients in the phenobarbital group received an initial 260 mg IV dose, and subsequent 130 mg IV doses repeated at physicians’ discretion. The comparison group received an initial lorazepam 2 mg IV dose, and subsequent 2 mg IV doses repeated at physicians’ discretion. Phenobarbital and lorazepam were similarly effective in treating mild or moderate alcohol withdrawal in the ED (no significant difference in change in CIWA scores between groups), with similar ED length of stay (267 min vs. 256 min, *p* = 0.8) and hospital admissions (48.0% vs. 84.2%, *p* = 0.8) [42].

#### Phenytoin alone

Three RCTs published before 1995 found no significant benefit to phenytoin compared to normal saline placebo in preventing seizure recurrence in the ED. [37, 38, 43]

## Discussion

### Summary of evidence

After an initial generalized seizure resulting from alcohol withdrawal, a single dose of IV lorazepam prevented seizure recurrence in the ED. [31] Sublingual lorazepam is more effective compared to placebo in reducing CIWA scores among patients in mild-to-moderate withdrawal [39]. There is no clear evidence that any one benzodiazepine is superior to another at improving withdrawal symptoms or preventing complications related to alcohol withdrawal syndrome [32].

Symptom-triggered protocols have been implemented in EDs with clinical decision units that can support longer stays, although patient outcomes have not been rigorously evaluated [33, 40]. One retrospective chart review suggested that a symptom-triggered protocol may decrease total doses of benzodiazepines administered, however, this finding would need to be replicated in a prospective, controlled study [40].

Existing studies do not show uniform evidence of benefit of phenobarbital (used alone, or in conjunction with symptom-triggered benzodiazepines) in multiple assessed outcomes: ICU admission, ED length of stay, and complications such as intubation [34–36, 41]. One RCT showed a 17.0% lower ICU admission (95% CI: 4.0 to 32.0%) among patients treated with IV phenobarbital combined with symptom-triggered lorazepam, but we assessed this study to be at high risk of bias [35].

Phenytoin is not effective versus normal saline placebo at preventing seizure recurrence related to alcohol withdrawal syndrome [37, 38, 43].

### Limitations

This review is limited by the overall poor quality of included studies, most of which were at high/serious risk of bias. We identified a lack of standardized definitions of alcohol withdrawal syndrome and severity among included studies. Studies also poorly reported detailed inclusion criteria, and/or clinical/patient information that would allow an interpretation of the populations most likely to benefit from each type of intervention.

Our use of rapid review methodology may increase the chance of inaccuracies in our study assessments vis-à-vis a formal systematic review. Nonetheless, we employed a systematic search strategy and our trained reviewers applied rigorous, prespecified criteria for inclusion, extraction, and risk of bias assessments, which strengthen our approach. Furthermore, our findings contribute more rigorous evidence compared to those previously published in expert opinion articles and narrative reviews. As most included studies were conducted in the United States and Canada, we are confident that our findings are likely generalizable within the North American context.

## Conclusions

### Comparison to previous studies

Our review highlights a paucity of studies evaluating the safety and efficacy of guideline-supported treatments for alcohol withdrawal syndrome (e.g., gabapentin and clonidine) when provided specifically in the ED setting [15, 16].

Unlike in the outpatient setting, ED patients generally present with more severe manifestations of withdrawal and are likely more medically complex. However, they may be more easily monitored, and medications and supportive treatments can be administered intravenously. Unlike in the inpatient setting, ED patients have undifferentiated presentations, are often being managed in high-volume settings (where care spaces, time for assessments, and clinical resources are stretched), and typically do not remain in the ED for more than 24 h. After ED treatment, clinicians must determine if patients are safe for discharge, or if they require hospital admission for further management.

Given the key differences between the ED compared to outpatient and inpatient contexts, there is a need for rigorous evidence evaluating the safety and effectiveness of ED-specific treatment approaches, and further guidance for risk stratification and resource allocation.

Finally, a previous review by Long et al. (2017), which summarized evidence from non-ED settings and non-interventional studies, proposed an algorithmic approach to alcohol withdrawal syndrome in the ED consisting of escalating doses of benzodiazepines, followed by phenobarbital, then propofol [20]. Our review supports the use of benzodiazepines as first-line treatment of severe alcohol withdrawal in the ED. However, our review of evidence from interventional studies performed in the ED does not provide sufficient evidence to recommend routine use of phenobarbital or propofol in ED treatment algorithms.

### Clinical implications

Benzodiazepines are the most evidence-based treatment for alcohol withdrawal treatment in the ED, especially for the prevention of alcohol withdrawal seizure recurrence. However, no clear evidence supports the use of one type of benzodiazepine over others. It is unclear if symptom-triggered protocols are effective for use in EDs, especially in those without attached observational units that can support longer stays. More evidence is needed to determine if phenobarbital, whether in combination with benzodiazepines or used alone, can be used safely and effectively for treatment of alcohol withdrawal syndrome in the ED, especially with regards to dosing, timing, and need for hospital admission. Phenytoin does not have evidence of effectiveness at preventing alcohol withdrawal seizures in the ED.

### Research implications

Given the rapidly changing landscape of alcohol-related ED visits during the COVID-19 pandemic, and the potential for new treatment strategies to quickly emerge, there is an urgent need in the near future for a full systematic review and evidence synthesis. Future studies should standardize definitions of alcohol withdrawal, outcome measures, and ascertainment of outcomes and adverse events; and distinguish between EDs with and without attached observational units; such that they can generate rigorous and generalizable evidence to guide ED management. Further studies are needed to evaluate symptom-triggered benzodiazepine protocols in the ED. Pharmacotherapies that have demonstrated benefit for treatment of alcohol withdrawal in other settings need to be evaluated in the ED setting before routine use.

## Data Availability

All data analyzed during this study are included in the published article.

## Appendix A Search strategy for MEDLINE (Ovid)

**Search Results**51 49 and 50 [Clinical Trials] (14)54 52 and 53 [Observ] (37)58 55 not 57 [Remaining] (35)**Date: ** May 11, 2020Database: Ovid MEDLINE(R) and Epub Ahead of Print, In-Process & Other Non-Indexed Citations, Daily and Versions(R) <1946 to May 08, 2020>Search Strategy:----------------------------------------------------------------1 emergency department?.ti,ab,kf. (90558)2 emergency room?.ti,ab,kf. (18922)3 emergency medicine.ti,ab,kf. (16075)4 (trauma adj2 (centre or center or room? or department?)).ti,ab,kf. (14027)5 or/1-4 (130470)6 *Emergency Medical Services/ (30007)7 *emergency service, hospital/ or *trauma centers/ (45024)8 *Emergency Services, Psychiatric/ (1847)9 *emergency medicine/ (10160)10 or/6-9 (83612)11 or/5,10 [Focused Emergency Department] (175175)12 *alcohol-related disorders/ (4101)13 *alcohol-induced disorders/ or *alcohol-induced disorders, nervous system/ or *alcohol withdrawal delirium/ or *alcohol withdrawal seizures/ (2016)14 *psychoses, alcoholic/ or *alcoholic intoxication/ or *alcoholism/ or *binge drinking/ (64734)15 alcohol withdrawal.ti,ab,kf. (3443)16 delirium tremens.ti,ab,kf. (1167)17 or/12-16 [Alcohol Withdrawal] (71222)18 11 and 17 [ED & Alcohol Withdrawal] (1422)19 exp benzodiazepines/ (65052)20 Benzodiazepine?.ti,ab,kw. (34309)21 exp barbiturates/ (53623)22 exp adrenergic alpha-2 receptor agonists/ (25402)23 exp "hypnotics and sedatives"/ (122536)24 exp antipsychotic agents/ (123243)25 exp Anticonvulsants/ (140816)26 baclofen/ (5580)27 Ethanol/tu, th [Therapeutic Use, Therapy] (2808)28 or/19-27 [Drug Therapy] (387290)29 and/11,17,28 [ED & Alcohol Disorders & Drug Therapy] (86)30 dt.fs. [Drug Therapy] (2201520)31 ad.fs. [Administration & Dose] (1402502)32 tu.fs. [Therapeutic Use] (2205294)33 Drug Therapy, Combination/ (164791)34 Dose-Response Relationship, Drug/ (401281)35 de.fs. [Drug Effects] (2966175)36 Drug Interactions/ (84883)37 or/30-36 [Pharmacological therapy] (5758470)38 and/11,17,37 [ED & Alcohol Disorders & Pharmacological therapy] (126)39 11 and 17 and (28 or 37) [ED & Alcohol Disorders & Clinical Intervention] (159)40 limit 39 to yr="1980 -Current" (157)41 limit 40 to English language (148)42 comment/ or editorial/ or letter/ or news/ (2022920)43 41 not 42 (141)44 animals/ not (animals/ and humans/) (4663791)45 43 not 44 (139)46 limit 45 to (systematic reviews pre 2019 or systematic reviews) (10)47 limit 45 to "reviews (best balance of sensitivity and specificity)" (26)48 or/46-47 [Reviews] (29)49 45 not 48 [Remaining] (110)50 exp clinical trial/ (858383)51 49 and 50 [Clinical Trials] (14)52 49 not 51 [Remaining] (96)53 retrospective studies/ or cohort studies/ or follow-up studies/ or prospective studies/ or controlled before-after studies/ or cross-sectional studies/ or Comparative Study/ (3689327)54 52 and 53 (37)55 52 not 54 [Remaining] (59)56 case reports/ (2095639)57 55 and 56 (24)58 55 not 57 [Remaining] (35)
