# Correction to: Prevention of alcohol withdrawal seizure recurrence and treatment of other alcohol withdrawal symptoms in the emergency department: a rapid review

**DOI:** 10.1186/s12873-022-00572-1

**Published:** 2022-01-31

**Authors:** Justin Jek-Kahn Koh, Madeline Malczewska, Mary M. Doyle-Waters, Jessica Moe

**Affiliations:** 1 Addiction Medicine Fellowship Program, British Columbia Centre for Substance Use, Vancouver, BC Canada; 2 Royal College Emergency Medicine Residency Program, Department of Emergency Medicine, College of Medicine, University of Saskatchewan, Saskatoon, SK Canada; 3 Faculty of Medicine, University of British Columbia, Vancouver, BC Canada; 4 Centre for Clinical Epidemiology and Evaluation, Vancouver Coastal Health Research Institute, Vancouver, BC Canada; 5 Department of Emergency Medicine, University of British Columbia, Vancouver, BC Canada; 6 Department of Emergency Medicine, Vancouver General Hospital, Vancouver, BC Canada; 7 British Columbia Centre for Disease Control, Vancouver, BC Canada

**Correction to: BMC Emerg Med 21, 131**

**https://doi.org/10.1186/s12873-021-00524-1**

The original article [1] contained a minor omission in co-author, Mary M. Doyle-Waters’ name which has since been corrected.
